# Supplementary material for: Using the VERT wearable device to monitor jumping loads in elite volleyball athletes
Source: PLoS One. 2021 Jan 22;16(1):e0245299. doi: 10.1371/journal.pone.0245299 (PMC7822237; doi:10.1371/journal.pone.0245299)
Supplement: Attachment — Submitted filename: Response to Reviewers 2.docx [file pone.0245299.s003.docx]

Reviewers' comments:

Reviewer #1: Dear authors, during the initial review I contacted the VERT company and even received the sensor specifications so you could have been a little more "brave" in disucssing the sensor specifications. Having said this, I think you have improved the manuscript and it is up to standard. I wish you had improved your presentation of the results a little to make a stronger point for your case. In the end your conclusion is: Shimmer is better in detecting peak accelerations which is a little "simple". Not every coach is really interested in the peak accelerations as they clearly depend on the sensor location, and sensor specs. Anyways, sorry to hear that your data collection was cut short due to the current global pandemic. Good luck with your future research!

We appreciate all of your feedback and attention to detail, which has improved the quality of our work.

Reviewer #2: The authors have adequately addressed my major concerns from the initial submission and the revised manuscript represents a marked improvement. I have a few further minor comments:

Thank you very much. Please see the responses to your specific comments below.

Line 115: Participants’ scores on the OSTRC-P should be reported with along with the other demographic measures in Table 1.

OSTRC-P scores are now included in Table 1.

Line 264: “on the ipad” – it would be better to say “on the measurement output produced by the Vert software”.

This has been amended.

Line 271: Change “preferred” to “chose”

This has been amended.
